# Raking of data from a large Australian cohort study improves generalisability of estimates of prevalence of health and behaviour characteristics and cancer incidence

**DOI:** 10.1186/s12874-022-01626-5

**Published:** 2022-05-14

**Authors:** Sarsha Yap, Qingwei Luo, Stephen Wade, Marianne Weber, Emily Banks, Karen Canfell, Dianne L. O’Connell, Julia Steinberg

**Affiliations:** 1 The Daffodil Centre, The University of Sydney, a joint venture with Cancer Council NSW, 153 Dowling St, Woolloomooloo, NSW 2011 Australia; 2 National Centre for Epidemiology and Population Health, Australian National University, Canberra, Australia; 3 School of Medicine and Public Health, University of Newcastle, Newcastle, NSW Australia

**Keywords:** Poststratification, Raking, Statistical weighting, Cohort studies, Health surveys, Representativeness, Nonresponse bias, Cancer incidence

## Abstract

**Background:**

Health surveys are commonly somewhat non-representative of their target population, potentially limiting the generalisability of prevalence estimates for health/behaviour characteristics and disease to the population. To reduce bias, weighting methods have been developed, though few studies have validated weighted survey estimates against generally accepted high-quality independent population benchmark estimates.

**Methods:**

We applied post-stratification and raking methods to the Australian 45 and Up Study using Census data and compared the resulting prevalence of characteristics to accepted population benchmark estimates and separately, the incidence rates of lung, colorectal, breast and prostate cancer to whole-of-population estimates using Standardised Incidence Ratios (SIRs).

**Results:**

The differences between 45 and Up Study and population benchmark estimates narrowed following sufficiently-informed raking, e.g. 13.6% unweighted prevalence of self-reported fair/poor overall health, compared to 17.0% after raking and 17.9% from a population benchmark estimate. Raking also improved generalisability of cancer incidence estimates. For example, unweighted 45 and Up Study versus whole-of-population SIRs were 0.700 (95%CI:0.574–0.848) for male lung cancer and 1.098 (95%CI:1.002–1.204) for prostate cancer, while estimated SIRs after sufficiently-informed raking were 0.828 (95%CI:0.684–0.998) and 1.019 (95%CI:0.926–1.121), respectively.

**Conclusion:**

Raking may be a useful tool for improving the generalisability of exposure prevalence and disease incidence from surveys to the population.

**Supplementary Information:**

The online version contains supplementary material available at 10.1186/s12874-022-01626-5.

## Background

Health surveys and cohort studies which link questionnaire data to other routinely collected health services information, are useful tools for measuring, understanding and tracking the health of populations. Such surveys and studies can assist in quantifying single or joint exposures, the occurrence of health conditions and interventions that promote or hinder health and may provide unique (or more timely) evidence on health. However, a surveyed sample is often not strictly representative of the target population of interest, defined as the population to which the estimates are meant to generalise [1]. For example, large-scale surveys and cohort studies, such as the Australian 45 and Up Study, the UK Biobank, United States (US) National Health Interview Survey, and All of Us, are non-representative by design [2–6], due to under- or over-representation of specific groups compared to the population as a whole. Representativeness is also impacted by selective non-response of those invited, which often leads to a healthier sample than the target population. Non-representativeness can limit the interpretation of exposure, disease incidence or prevalence estimates, particularly, the generalisability of these estimates to the corresponding population.

There are several weighting methods to improve the representativeness of survey-derived estimates to the target population, such as post-stratification, and emerging approaches including raking, quasi-randomisation and other model-based weighting approaches [7–10]. Post-stratification is commonly used to assign weights to participants so that the weighted joint distribution of selected characteristics in the sample matches that in the target population [11]. As this approach requires cross-tabulating selected characteristics to form subgroups, the number of characteristics that can be used is limited when small counts in some subgroups result in unstable weights. More recently, raking has been used to incorporate more characteristics into the weights. Raking is a proportional iterative procedure which adjusts the sample’s weighted marginal distributions for selected characteristics to match those of the target population [12]. Weighting is often applied to surveys, however, few studies have validated the weighted estimates of behaviour and health characteristics against other representative population estimates. Additionally, to the best of our knowledge, no studies have shown how weighted cancer incidence rates for a sample compare to the corresponding whole-of-population rates.

The 45 and Up Study collected a range of health, demographic, and lifestyle information from over 260,000 New South Wales (NSW) residents aged 45 and over [2], recruited 2006–2009. It is the largest longitudinal study of ageing in Australia, with over 400 publications to date [13]. Linkage of the 45 and Up Study questionnaire data to routinely collected health information such as cancer registrations, has provided rich insights on the health of the population. To examine the health of some population groups, the study deliberately oversampled the elderly and people living in rural areas and hence, is non-representative of the general NSW or Australian populations. However, the overall sample includes a wide range of population groups, making it an excellent candidate for assessing the impact of weighting on study estimates. Our aim was to apply post-stratification and raking methods to the 45 and Up Study’s baseline data and compare the resulting weighted: (1) distributions of selected characteristics to the Australian Census and independent high-quality population benchmark estimates for NSW and Australia; and (2) incidence of lung, colorectal, breast and prostate cancers from the 45 and Up Study to that of the whole of NSW and Australia.

## Methods

### 45 and Up Study data

The Sax Institute’s 45 and Up Study cohort comprises 267,153 people from NSW, Australia, recruited between January 2006 and December 2009. Participants aged ≥ 45 years were randomly sampled from the Services Australia (formerly the Australian Government Department of Human Services) Medicare enrolment database that has near-complete coverage of the population. People living in remote and rural areas and those aged ≥ 80 years were oversampled. Overall, the response rate was ~ 18% and the cohort represents ~ 11% of the NSW population aged ≥ 45 years. Participants self-completed a postal questionnaire at recruitment, which included health, socio-demographic and past medical history information. Further details are described elsewhere [2].

Baseline data were linked to the NSW Cancer Registry (NSWCR; 01-January-1994 to 31-December-2013), which contains all notifications of cancer diagnosed in NSW, to ascertain primary incident cancers of the lung (ICD-10 classification code: C33-C34), colorectum (C18-C20), prostate (C61) and female breast (C50). Cases with a record prior to or at recruitment were excluded. Additionally, we linked to NSW Registry of Births, Deaths and Marriages (RBDM; 01-February-2006 to 31-December-2013) to ascertain deaths that occurred before the end of follow-up (i.e., 31-December-2013) required for calculating person-years at risk. Data were sourced from the Cancer Institute NSW and NSW Ministry of Health and were probabilistically linked by the Centre for Health Record Linkage using a best practice approach to linkage while preserving privacy [14]. The probabilistic matching process is known to be highly accurate (false-positive and false-negative rates of ~ 0.5%) [15]. All data were accessed using the Secure Unified Research Environment (SURE).

The conduct of the 45 and Up Study was approved by the University of New South Wales Human Research Ethics Committee. The NSW Population and Health Services Research Ethics Committee approved the record linkage and analysis of the 45 and Up Study data (approval number 2014/08/551).

### Population data used for developing weights

#### The Census of Population and Housing Survey data

The Census is a compulsory survey of all people in Australia, conducted by the Australian Bureau of Statistics (ABS) every five years, and provides demographic, socioeconomic and housing characteristics of the entire population. Data for people aged ≥ 45 years from the 2006 Census, the closest in time to recruitment of the 45 and Up Study sample, were obtained using ABS online Table Builder Basic [16]. We considered all characteristics in the Census that were highly comparable to those in the 45 and Up Study’s baseline questionnaire (Additional file 1, Table A). This identified the seven characteristics (sex, 5-year age group, place of residence (coded using the Accessibility and Remoteness Index of Australia [ARIA]), education, region of birth, language other than English spoken at home and marital status) which were then considered further for inclusion in the weights.

### Surveys used to compare health characteristics and behaviours

As many health and behaviour characteristics are not included in the Census, we compared the estimated prevalence of these from the 45 and Up Study to those from two independent population benchmarks.

#### National Drug Strategy Household Survey (NDSHS) data

The NDSHS is conducted by the Australian Institute of Health and Welfare (AIHW) every three years and provides information on alcohol, tobacco and illicit drug use for a representative sample of the Australian population (see Additional file 1, Table B for characteristics used in this study) [17, 18]. To ensure compatibility with the 45 and Up Study’s mode of data collection, we included data collected using self-completed questionnaires (85% and 100% of all survey participants in 2007 and 2010, respectively). Data for participants aged ≥ 45 years from the 2007 (*n* = 12,470) and 2010 (*n* = 14,388) surveys were used, with overall response rates of 54% and 51%, respectively. Data from each survey were weighted using weights supplied with the survey information so that the sample was approximately representative of the Australian population in terms of age, sex, place of residence and household size.

#### Australian National Health Survey (ANHS) data

The ANHS is a household survey conducted by the ABS every three years which provides health information for a sample of the Australian population [19]. Data from the 2007 survey were obtained using the Remote Access Data Laboratory [20]. There were 15,800 households randomly sampled (91% response rate), and 8,531 people aged ≥ 45 years were interviewed in person. We identified 17 characteristics from the ANHS questionnaire that were comparable to items in the 45 and Up Study’s baseline questionnaire (Additional file 1, Table B). Weighted frequencies for these characteristics in the ANHS were calculated using the person weights provided in the dataset, which adjusted for the probability of a person being selected and were calibrated so that the proportions in the sample aligned with those in the Australian population for sex, age group and place of usual residence.

### Population-wide cancer incidence data

The total numbers of people by sex and 5-year age group for the NSW and Australian population were obtained from the ABS [21].

We obtained the NSW-wide numbers of incident primary lung, colorectum, prostate and female breast cancers by sex and 5-year age group from the NSWCR for 01-January-2009 to 31-December-2013, using the same ICD-10 codes as above. To match the inclusion criteria used for the 45 and Up Study, NSWCR cases were excluded if they were diagnosed with multiple primary cancers, secondary cancers or who were notified to the NSWCR through death certificate only. The NSW Population and Health Services Research Ethics Committee approved the analysis of cancer incidence data for all of NSW (Reference: HREC/09/CIPHS/16).

We did not have access to primary cancer incidence data for the whole of Australia with equivalent inclusion criteria to those for the 45 and Up Study cohort. However, age-standardised NSW cancer incidence rates for lung, colorectal, prostate and breast cancers are almost identical to the Australian rates when equivalent inclusion criteria are used as reported in Cancer Data in Australia by the AIHW for 1982–2016 (Additional file 2 with all rates standardised to the Australian population in 2001) [22]. Consequently, we used the NSWCR data as a proxy for the Australian national rates.

### Statistical analyses

All analyses were conducted in SAS 9.4 and STATA (release 16.1. College Station, TX: Stata Corporation; 2019).

### Weighting methods

We applied post-stratification and raking methods to data from the 45 and Up Study, to derive weights matching the distribution of demographic data in the 2006 Australian Census for the NSW and Australian populations. We used both a ‘full’ and ‘basic’ set of characteristics to construct separate raking weights, and the basic set to construct post-stratification weights.

#### Raking

Seven demographic characteristics (listed in Table 1) were selected to create two raked weights for the 45 and Up Study (‘full raking’), one each for the NSW and Australian populations. Another set of weights were created separately for the NSW and Australian populations using ‘basic raking’ with sex, 5-year age group and place of residence only. Participants from the sample were excluded (*n* = 11,788) if they had missing values for any of the characteristics used to construct the weights. For each estimated weight, values outside of the median plus six times the interquartile range (IQR) were trimmed to remove extreme outliers. We used the STATA ipfraking package [12] to calculate the raked weights. The Additional file 3 (‘Development of raking weights’) includes a step-by-step description of the method.

**Table 1:** 45 and Up Study participants’ characteristics (2006–2009) used in fully raked weighting and comparison with Census data for the NSW and Australian populations

Weighting characteristic	45 and Up Study unweighted (*N* = 255,365)		Census 2006 NSW (*N* = 2,529,664)	45 and Up Study weighted to NSW^a^ (full raking)	Census 2006 Australia (*N* = 7,599,570)	45 and Up Study weighted to Australia^b^ (full raking)
	**n**	**% (95% CI)**	**%**	**% (95% CI)**	**%**	**% (95% CI)**
**Sex**						
Male	117,969	46.2 (46.0, 46.4)	47.8	47.8 (47.5, 48.1)	47.9	47.9 (47.6, 48.2)
Female	137,396	53.8 (53.6, 54.0)	52.2	52.2 (51.9, 52.5)	52.1	52.1 (51.8, 52.4)
**Age group (years)**						
45–49	33,711	13.2 (13.1, 13.3)	18.8	18.8 (18.6, 19.0)	19.0	19.0 (18.8, 19.3)
50–54	41,504	16.3 (16.1, 16.4)	17.0	17.0 (16.8, 17.2)	17.3	17.3 (17.1, 17.5)
55–59	43,865	17.2 (17.0, 17.3)	15.9	15.9 (15.7, 16.1)	16.2	16.2 (16.1, 16.4)
60–64	38,710	15.2 (15.0, 15.3)	12.6	12.6 (12.4, 12.7)	12.6	12.6 (12.5, 12.8)
65–69	32,235	12.6 (12.5, 12.8)	10.1	10.1 (9.9, 10.2)	10.0	10.0 (9.8, 10.1)
70–74	23,168	9.1 (9.0, 9.2)	8.3	8.3 (8.2, 8.5)	8.1	8.1 (8.0, 8.2)
75–79	16,949	6.6 (6.5, 6.7)	7.4	7.4 (7.3, 7.6)	7.2	7.2 (7.0, 7.3)
80–84	17,621	6.9 (6.8, 7.0)	5.6	5.6 (5.5, 5.7)	5.3	5.3 (5.2, 5.4)
85 +	7,602	3.0 (2.9, 3.0)	4.4	4.4 (4.3, 4.5)	4.2	4.2 (4.1, 4.4)
**Place of residence (ARIA)**						
Major City	135,389	53.0 (52.8, 53.2)	69.0	69.0 (68.6, 69.3)	66.1	66.2 (65.9, 66.6)
Inner Regional	90,786	35.6 (35.4, 35.7)	22.8	22.8 (22.7, 23.0)	21.7	21.8 (21.6, 21.9)
Outer Regional	26,704	10.5 (10.3, 10.6)	7.7	7.7 (7.6, 7.8)	10.1	10.1 (9.9, 10.2)
Remote/Very Remote	2,486	1.0 (0.9, 1.0)	0.6	0.6 (0.5, 0.6)	1.9	1.9 (1.8, 2.0)
**Educational attainment**						
No School Certificate	30,327	11.9 (11.8, 12.0)	22.8	22.8 (22.5, 23.1)	22.3	22.3 (22.0, 22.6)
School Certificate	57,213	22.4 (22.2, 22.6)	23.2	23.2 (23.0, 23.4)	24.4	24.4 (24.2, 24.7)
Trade/Certificate/Diploma	82,527	32.3 (32.1, 32.5)	28.0	28.0 (27.8, 28.2)	27.7	27.7 (27.5, 27.9)
Higher School Certificate	25,364	9.9 (9.8, 10.0)	12.4	12.4 (12.2, 12.6)	12.7	12.7 (12.5, 12.9)
University degree or higher	59,934	23.5 (23.3, 23.6)	13.7	13.7 (13.6, 13.8)	12.8	12.8 (12.7, 13.0)
**Region of birth**						
Australia	193,250	75.7 (75.5, 75.8)	66.0	66.0 (65.7, 66.2)	66.4	66.4 (66.1, 66.6)
NZ and Oceania	5,760	2.3 (2.2, 2.3)	2.5	2.5 (2.5, 2.6)	2.7	2.7 (2.6, 2.8)
Asia	8,901	3.5 (3.4, 3.6)	8.1	8.1 (7.9, 8.2)	6.0	6.0 (5.9, 6.2)
UK and Ireland	25,270	9.9 (9.8, 10.0)	8.0	8.0 (7.9, 8.1)	10.4	10.4 (10.2, 10.5)
Europe	14,891	5.8 (5.7, 5.9)	10.4	10.4 (10.2, 10.6)	10.9	10.9 (10.7, 11.1)
Other	7,293	2.9 (2.8, 2.9)	5.1	5.1 (5.0, 5.2)	3.6	3.6 (3.5, 3.7)
**Language other than English**						
No	231,220	90.5 (90.4, 90.7)	80.2	80.3 (80.1, 80.6)	83.5	83.5 (83.2, 83.7)
Yes	24,145	9.5 (9.3, 9.6)	19.8	19.7 (19.4, 20.0)	16.5	16.5 (16.3, 16.7)
**Marital status**						
Never married	15,975	6.3 (6.2, 6.3)	8.4	8.4 (8.2, 8.5)	7.9	7.9 (7.7, 8.0)
Widowed	22,040	8.6 (8.5, 8.7)	12.5	12.5 (12.3, 12.7)	12.0	12.0 (11.8, 12.2)
Divorced	19,059	7.5 (7.4, 7.6)	11.9	11.9 (11.7, 12.1)	12.3	12.3 (12.1, 12.5)
Separated	7,124	2.8 (2.7, 2.9)	3.8	3.8 (3.7, 3.9)	3.8	3.8 (3.7, 3.9)
Married	191,167	74.9 (74.7, 75.0)	63.5	63.5 (63.2, 63.7)	64.0	64.0 (63.8, 64.2)

^a^Estimates after full raking based on all characteristics listed in Table 1 and matching to the ABS Census 2006 data (restricted to the NSW population)

^b^Estimates after full raking based on all characteristics listed in Table 1 and matching to the ABS Census 2006 data for the whole Australian population. *ABS* Australian Bureau of Statistics, *ARIA* Accessibility and Remoteness Index of Australia, *CI* Confidence Intervals, *NSW* New South Wales, *NZ* New Zealand, *UK* United Kingdom

#### Post-stratification weighting

We created two post-stratification weights to match the NSW and Australian populations separately, using the same characteristics as for ‘basic raking’ (with a total of 2 × 9 × 4 = 72 combinations).

### Comparison of the prevalence of health characteristics and behaviours

To establish whether raking and post-stratification weighting improved the representativeness of the 45 and Up Study cohort, we compared distributions of participants’ health and lifestyle characteristics, which were not included in the raking weights, to those in the NDSHS and ANHS (listed in Table 2). All NDSHS and ANHS questionnaire items were examined for similarity to those in the 45 and Up Study. Six characteristics in both surveys were identified as moderately or highly comparable to the 45 and Up Study.

**Table 2:** 45 and Up Study participants’ socioeconomic, health and lifestyle characteristics (2006–2009) before and after applying fully raked weights, compared to those in the NDSHS and ANHS

Characteristic	45 and Up Study unweighted (*N* = 255,365)		45 and Up Study weighted to NSW^a^ (full raking)	NDSHS NSW^b^ (*N* = 7,963)	ANHS NSW^c^ (*N* = 1,625)	45 and Up Study weighted to Australia^d^ (full raking)	NDSHS Australia^e^ (*N* = 26,858)	ANHS Australia^f^ (*N* = 8,531)
	**N**	**% (95% CI)**	**% (95% CI)**	**% (95% CI)**	**%**	**% (95% CI)**	**% (95% CI)**	**%**
**Body Mass Index (kg/m**^**2**^**)**								
Underweight (< 18.5)	3,216	1.3 (1.2, 1.3)	1.4 (1.4, 1.5)	1.6 (1.2, 2.0)	0.7	1.4 (1.3, 1.4)	1.5 (1.3, 1.7)	0.7
Normal Range (18.5 to < 25)	87,198	34.1 (34.0, 34.3)	33.9 (33.6, 34.1)	33.2 (31.6, 34.9)	22.4	33.3 (33.1, 33.6)	31.8 (30.9, 32.7)	20.5
Overweight (25 to < 30)	93,476	36.6 (36.4, 36.8)	35.6 (35.3, 35.8)	34.6 (33.0, 36.2)	29.0	35.8 (35.6, 36.1)	35.6 (34.7, 36.5)	28.3
Obese (≥ 30)	52,847	20.7 (20.5, 20.9)	21.5 (21.3, 21.7)	22.8 (21.4, 24.3)	21.5	21.9 (21.7, 22.1)	23.4 (22.6, 24.2)	21.1
Missing	18,628	7.3 (7.2, 7.4)	7.6 (7.5, 7.8)	7.8 (6.9, 8.7)	26.4	7.6 (7.5, 7.7)	7.7 (7.2, 8.2)	29.4
**Employment status**								
Employed	129,654	50.8 (50.6, 51.0)	50.1 (49.8, 50.4)	44.6 (42.8, 46.4)	49.6	51.1 (50.8, 51.4)	46.3 (45.3, 47.3)	51.9
Unemployed	5,685	2.2 (2.2, 2.3)	3.3 (3.2, 3.4)	2.3 (1.7, 2.9)	1.4	3.2 (3.1, 3.3)	2.1 (1.8, 2.4)	1.0
Not in the labour force	115,845	45.4 (45.2, 45.6)	44.4 (44.1, 44.7)	47.2 (45.4, 49.0)	49.0	43.6 (43.3, 43.8)	46.0 (45.0, 47.0)	47.0
Missing	4,181	1.6 (1.6, 1.7)	2.2 (2.1, 2.2)	5.9 (5.1, 6.8)		2.1 (2.0, 2.2)	5.6 (5.2, 6.1)	
**K10 distress scale**								
Well (0 to 19)	200,014	78.3 (78.2, 78.5)	74.1 (73.8, 74.3)	87.5 (86.2, 88.8)	82.9	74.6 (74.3, 74.9)	87.8 (87.1, 88.5)	83.9
Mild (20 to 24)	14,655	5.7 (5.6, 5.8)	6.7 (6.6, 6.9)	6.6 (5.7, 7.6)	8.6	6.7 (6.6, 6.8)	7.0 (6.4, 7.5)	8.2
Moderate (25 to 29)	4,788	1.9 (1.8, 1.9)	2.5 (2.4, 2.5)	3.0 (2.4, 3.7)	3.9	2.4 (2.3, 2.5)	2.7 (2.4, 3.0)	4.0
Severe (30 to 50)	4,664	1.8 (1.8, 1.9)	2.7 (2.6, 2.8)	2.3 (1.8, 2.9)	4.4	2.7 (2.6, 2.8)	2.1 (1.8, 2.4)	3.8
Missing	31,244	12.2 (12.1, 12.4)	14.0 (13.8, 14.2)	0.5 (0.3, 0.7)	0.2	13.6 (13.5, 13.8)	0.5 (0.3, 0.6)	0.1
**Overall health**								
Excellent	37,394	14.6 (14.5, 14.8)	12.6 (12.5, 12.8)	10.8 (9.7, 12.0)	15.8	12.9 (12.7, 13.0)	11.2 (10.5, 11.8)	15.7
Very Good	91,525	35.8 (35.7, 36.0)	32.4 (32.2, 32.6)	33.0 (31.3, 34.7)	30.8	32.9 (32.6, 33.1)	33.7 (32.7, 34.6)	31.2
Good	83,318	32.6 (32.4, 32.8)	34.0 (33.8, 34.3)	37.1 (35.4, 38.9)	30.4	33.8 (33.6, 34.1)	36.5 (35.6, 37.5)	30.6
Fair	29,281	11.5 (11.3, 11.6)	14.0 (13.8, 14.2)	14.8 (13.6, 16.1)	16.8	13.7 (13.5, 13.9)	14.5 (13.8, 15.2)	15.7
Poor	5,327	2.1 (2.0, 2.1)	3.0 (2.9, 3.1)	3.1 (2.5, 3.8)	6.3	2.9 (2.8, 3.0)	2.9 (2.6, 3.2)	6.9
Missing	8,520	3.3 (3.3, 3.4)	4.0 (3.9, 4.1)	1.1 (0.7, 1.4)		3.9 (3.8, 4.0)	1.2 (1.0, 1.4)	
**Smoking status at baseline**								
Current regular smoker	18,265	7.2 (7.1, 7.3)	9.5 (9.3, 9.7)	13.9 (12.7, 15.2)	17.6	9.7 (9.6, 9.9)	13.9 (12.7, 15.2)	16.0
Former regular smoker	91,398	35.8 (35.6, 36.0)	35.1 (34.8, 35.3)	35.3 (33.6, 37.0)	36.2	35.8 (35.5, 36.0)	35.3 (33.6, 37.0)	37.4
Never regular smoker	145,609	57.0 (56.8, 57.2)	55.4 (55.1, 55.7)	50.6 (48.8, 52.4)	46.2	54.5 (54.2, 54.7)	50.6 (48.8, 52.4)	46.6
Missing	93	0.0 (0.0, 0.0)	0.0 (0.0, 0.1)	0.2 (0.0, 0.3)		0.0 (0.0, 0.1)	0.2 (0.0, 0.3)	
**Smoking duration**								
< 6 years	6,549	2.6 (2.5, 2.6)	2.3 (2.2, 2.3)	4.2 (3.4, 4.9)	4.1	2.3 (2.3, 2.4)	4.0 (3.6, 4.4)	4.0
6–10 years	11,426	4.5 (4.4, 4.6)	4.0 (4.0, 4.1)	4.6 (3.9, 5.4)	3.9	4.2 (4.1, 4.3)	4.6 (4.2, 5.0)	4.2
11–19 years	20,306	8.0 (7.8, 8.1)	7.5 (7.4, 7.6)	7.1 (6.2, 8.0)	6.9	7.7 (7.6, 7.8)	7.2 (6.7, 7.7)	7.9
20–29 years	22,509	8.8 (8.7, 8.9)	9.1 (9.0, 9.3)	8.8 (7.8, 9.8)	10.5	9.3 (9.1, 9.4)	9.2 (8.6, 9.8)	10.4
30–39 years	23,867	9.3 (9.2, 9.5)	10.8 (10.6, 10.9)	10.9 (9.8, 12.0)	13.1	11.1 (10.9, 11.2)	11.7 (11.0, 12.3)	13.0
40 + years	17,949	7.0 (6.9, 7.1)	7.6 (7.5, 7.7)	8.1 (7.2, 9.0)	12.3	7.7 (7.5, 7.8)	8.3 (7.8, 8.8)	10.7
Not applicable or missing	152,759	59.8 (59.6, 60.0)	58.7 (58.4, 59.0)	56.2 (54.4, 58.0)	49.2	57.8 (57.5, 58.1)	55.0 (54.0, 56.0)	49.8

*ABS* Australian Bureau of Statistics, *ANHS* Australian National Health Survey, *CI* Confidence Intervals, *K10* Kessler psychological distress scale, *NDSHS* National Drug Strategy Household Survey, *NSW* New South Wales

^a^Estimates after full raking based on all characteristics listed in Table 1 and matching to the ABS Census 2006 data (restricted to the NSW population)

^b^Weighted using the absolute person weight provided in the NDSHS 2007 and 2010 datasets, including NSW participants only. This weight adjusts the probability of selection based on sex, age, place of residence, household size and survey delivery method

^c^Weighted using the person weight provided in the ANHS 2007 dataset, including NSW participants only. This weight adjusts the probability of selection based on sex, age and place of residence

^d^Estimates after full raking based on all characteristics listed in Table 1 and matching to the ABS Census 2006 data for the whole Australian population

^e^Weighted using the absolute person weight provided in the NDSHS 2007 and 2010 datasets, including all participants. This weight adjusts the probability of selection based on sex, age, place of residence, household size and survey delivery method

^f^Weighted using the person weight provided in the ANHS 2007 dataset, including all participants. This weight adjusts the probability of selection based on sex, age and place of residence

The unweighted and weighted prevalence of each characteristic was estimated with 95% confidence intervals (95%CIs) in the 45 and Up Study using the SAS surveyfreq procedure. Weighted percentages and 95%CIs for these characteristics in the NDSHS were generated using weights provided in the dataset and the STATA `svy` function. For characteristics that were available from both NDSHS datasets, we estimated the prevalence separately for 2007 and 2010, and as these were similar for all characteristics, we used the averaged weighted estimates. We additionally compared estimates for eight characteristics in the 45 and Up Study to those in the ANHS that were not available in the NDSHS, including private health insurance, Department of Veterans’ Affairs (DVA) white or gold healthcare benefits cards, ever diagnosed with asthma or diabetes, number of alcoholic drinks per week, fruit and vegetable consumption and the main type of milk consumed. However, the ANHS data available to us did not include confidence intervals.

To summarise the overall effectiveness of post-stratification, basic raking and full raking in reducing the absolute difference between 45 and Up Study weighted estimates and population benchmark estimates, we calculated four measures based on all characteristics together: 1) the number of categories with overlapping 95% confidence intervals for the NDSHS population estimates and the weighted and unweighted 45 and Up Study estimates; 2) the number of categories for which the population benchmark estimates were within the 95% confidence intervals of the weighted and unweighted 45 and Up Study estimates; 3) the number of categories for which the weighted 45 and Up Study point estimates moved closer to the population benchmark estimates relative to the corresponding unweighted estimates; and 4) the median and interquartile range (IQR) for the absolute difference between the population benchmark estimates and the weighted and unweighted 45 and Up Study estimates.

### Comparison of cancer incidence rates

We compared the unweighted and weighted cancer incidence in the 45 and Up Study to that for the NSW and Australian populations, separately for males, females, and each cancer type. We used indirect standardisation to estimate the standardised incidence ratio (SIR) by dividing the unweighted or weighted observed number of cancer cases (O) by the expected number (E) in the 45 and Up Study [23]. A detailed description of the method can be found in the Additional file 4 (‘Calculation of standardised incidence ratios’). The expected numbers of new cancer cases were determined using the sex-age-specific incidence rates for the reference population multiplied by the unweighted or weighted person-years at risk in the study cohort. As noted above, the calculations for Australia used the NSW incidence rates as a proxy for Australian rates, and the 45 and Up Study sample weighted to the Australian population.

We calculated the confidence intervals for the SIRs using the Fieller-based method (see Additional file 4 for details). As the 45 and Up Study deliberately over-sampled individuals ≥ 80 years old, we used a second approach to verify the robustness of results (see Additional file 4). The weighted observed and expected numbers of cases were estimated using the STATA `svy` function [24].

## Results

All analyses included 255,365 45 and Up Study participants with non-missing values for all seven characteristics used for weighting.

### Full raking adjusts the cohort’s characteristics used for weighting almost precisely to the target populations

Without weighting, a higher proportion of 45 and Up Study participants had a university degree, were married, born in Australia, and spoke only English at home compared to the NSW (*N* = 2,529,664) and Australian populations (*N* = 7,599,570) aged ≥ 45 years (Table 1). Reflecting the sampling scheme, a smaller proportion of participants lived in a major city. After full raking, the weighted percentages for all characteristics almost exactly matched those of the NSW and Australian populations. After basic raking and post-stratification, weighted percentages for age, sex and place of residence were equivalent to those in the target population. However, these latter two approaches did not rectify the over-representation of those with higher educational attainment, who were married, born in Australia or spoke only English (Additional file 5).

### Full raking improves representativeness of the 45 and Up Study cohort on several health and lifestyle characteristics

**Fig. 1:**
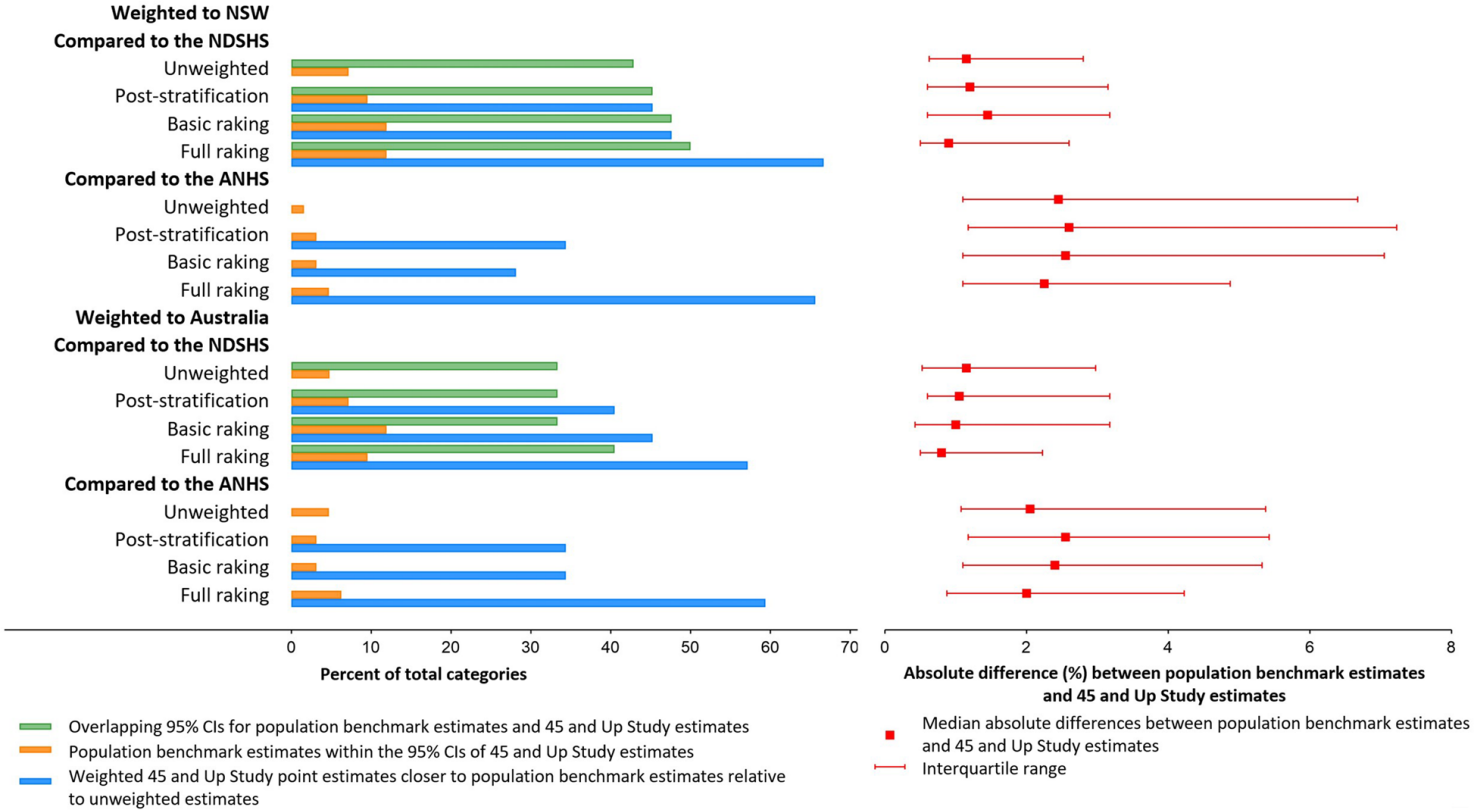
Summary measures of weighting effectiveness for post-stratification, basic raking and full raking. The summary measures are based on all 18 characteristics shown in Additional file 6, with a total of 68 categories (excluding “missing”), of which 64 and 42 categories are included in the comparisons with the ANHS and NDSHS population benchmarks, respectively. The numbers of categories with overlapping 95% confidence intervals for the ANHS estimates and the unweighted or weighted 45 and Up Study estimates were not calculated, as the ANHS data used in this work did not include confidence intervals. *CI* confidence interval, *NDSHS* National Drug Strategy Household Survey, *ANHS* Australian National Health Survey.

Overall, the 45 and Up Study was compared to the NDSHS on ten characteristics with a total of 42 categories (excluding “missing”; Fig. 1 and Additional file 6). The number of categories with overlapping 95% confidence intervals for the NDSHS estimates and the unweighted estimates was 18 (42.9%), while the number with overlapping 95% confidence intervals between the NDSHS estimates and weighted estimates for post-stratification, basic raking and full raking were 19 (45.2%), 20 (47.6%) and 21 (50.0%), respectively. The number of categories for which the NDSHS estimates were within the 95% confidence intervals for the unweighted estimates was 3 (7.1%), compared to 4 (9.5%), 5 (11.9%) and 5 (11.9%) for post-stratification, basic raking and full raking, respectively. Of the 42 categories, post-stratification, basic raking and full raking moved the 45 and Up Study weighted estimates closer to the NDSHS NSW estimates (relative to the unweighted estimates) for 19 (45.2%), 20 (47.6%) and 28 (66.7%) categories, respectively. The median absolute difference between the population benchmark estimates and unweighted estimates was 1.1% (IQR 0.6%-2.8%), while the median absolute difference between the population benchmark estimates and weighted estimates for post-stratification, basic raking and full raking was 1.2% (0.6%-3.2%), 1.5% (0.6%-3.2%) and 0.9% (0.5%-2.6%), respectively. Similar patterns were observed when comparing the NDSHS estimates for Australia to unweighted and weighted 45 and Up Study estimates for Australia.

Without weighting, compared to the NDSHS, larger proportions of 45 and Up Study participants were overweight (+ 2%), had very good overall health (+ 2.8%) and were never smokers (+ 6.4%). By contrast, there were smaller proportions who were not in the labour force (-1.8%), had fair or poor overall health (-3.3% and -1%), were current smokers (-6.7%) and smoked 30 + years (-2.7%). Results were similar when the 45 and Up Study data were compared to the ANHS. For all characteristics except the Kessler-10 psychological distress scale (K10), full raking produced estimates that were closer to those from the NDSHS, e.g. prevalence differences after full raking reduced to + 4.8% for never smokers, -0.8% for fair and -0.1% for poor overall health, and -4.4% for current smokers. As there was a much higher proportion of missing responses in the 45 and Up Study for the K10 compared to the other surveys, resulting in under-representation in all other K10 categories, it was not possible to assess the impact of weighting for this characteristic.

The 45 and Up Study was compared to the ANHS on 17 characteristics with 64 categories (excluding “missing”; Fig. 1 and Additional file 6). The number of categories for which the ANHS estimates were within the 95% confidence intervals for the unweighted estimates was 1 (1.6%), compared to 2 (3.1%), 2 (3.1%) and 3 (4.7%) for post-stratification, basic raking and full raking, respectively. Of the 64 categories, post-stratification, basic raking and full raking moved the 45 and Up Study weighted estimates closer to the ANHS NSW estimates for 22 (34.4%), 18 (28.1%) and 42 (65.6%) categories, respectively. The median absolute difference between the population benchmark estimates and unweighted estimates was 2.5% (IQR 1.1%-6.7%), while the median absolute difference between population benchmark estimates and weighted estimates for post-stratification, basic raking and full raking was 2.6% (1.2%-7.2%), 2.5% (1.1%-7.1%) and 2.3% (1.1%-4.9%), respectively. Similar patterns were observed when comparing the ANHS estimates for Australia to unweighted and weighted 45 and Up Study estimates for Australia.

For the eight additional characteristics in the ANHS, without weighting, larger proportions of 45 and Up Study participants had private health insurance (+ 7.7%), moderate alcohol consumption (3.5–14 alcoholic drinks per week; + 13.5%) and ate > 5 serves of vegetables per day (+ 20.4%)(Additional file 6). There were smaller proportions with self-reported diabetes (-4.8%), who were non-drinkers (-9.2%), ate < 2 serves of fruit per day (-5.2%) and drank whole milk (-10.6%). Again, after full raking, estimates were more similar to those from the ANHS for five of eight characteristics, including private health insurance (+ 0.7%) and non-drinkers (-4.5%).

For most characteristics, post-stratification or basic raking resulted in weighted estimates that were very similar to the unweighted estimates, and hence did not reduce the differences between the 45 and Up Study and the NDSHS or ANHS (Additional file 6). For selected characteristics such as household income and private health insurance, these two approaches further increased the over-representation of the affluent groups.

### Full raking improves representativeness of cancer incidence in the 45 and Up Study cohort

**Fig. 2:**
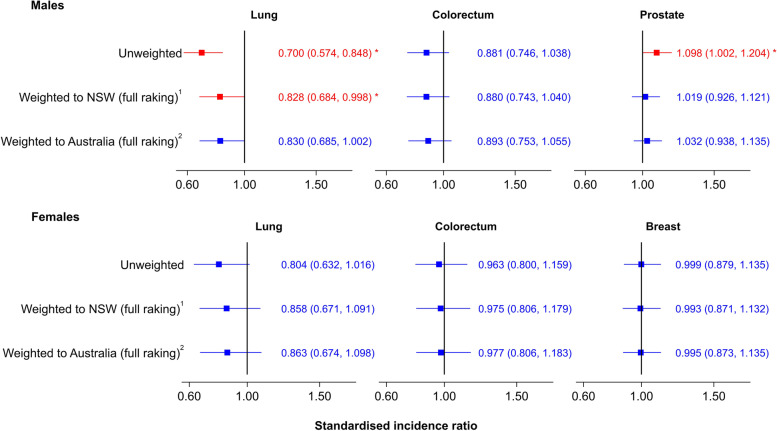
Unweighted and weighted Standardised Incidence Ratios (SIRs) for cancers of the lung, colorectum, prostate and breast for the 45 and Up Study cohort compared to the NSW and Australian populations, using NSW Cancer Registry data (2009–2013). 1 Estimates after full raking based on all characteristics listed in Table 1 and matching to the ABS Census 2006 data (restricted to the NSW population). 2 Estimates after full raking based on all characteristics listed in Table 1 and matching to the ABS Census 2006 data for the whole Australian population. * Statistically significant at 5% level

Without weighting, there was lower incidence of lung cancer for males (SIR = 0.700, 95%CI:0.574–0.848) and higher incidence of prostate cancer (SIR = 1.098, 95%CI:1.002–1.204) in the 45 and Up Study compared to the NSW population (Fig. 2), with similar incidence of colorectal cancer in both sexes, and breast cancer and lung cancer in females. After full raking, the incidence of lung and prostate cancers for males in the 45 and Up Study was more comparable to that for NSW (SIR = 0.828, 95%CI:0.684–0.998 and SIR = 1.019, 95%CI:0.926–1.121, respectively) and Australia (SIR = 0.830, 95%CI:0.685–1.002 and SIR = 1.032, 95%CI:0.938–1.135, respectively). By contrast, weighting using post-stratification or basic raking to both the NSW and Australian populations was less effective in reducing differences in incidence (Additional file 7).

## Discussion

Full raking to weight the participants in a large Australian cohort provided more generalisable estimates of the prevalence of key health and sociodemographic characteristics and of cancer incidence. Full raking shifted the 45 and Up Study estimates closer to those high-quality population benchmark estimates for the NSW and Australian populations, and where unweighted estimates were already similar to those for the target population, weighted and unweighted estimates remained similar. Our results highlight the importance of selecting appropriate characteristics to obtain the weights: full raking including all seven characteristics was more effective than basic raking or post-stratification using sex, age and place of residence only. Basic raking and post-stratification produced similar results: both improved the representativeness of estimates for characteristics used in the weights, but did not substantially improve representativeness of estimates for the majority of other characteristics. These results also highlight the advantage of the raking method, which can incorporate more characteristics into the weights compared to the more commonly used post-stratification method. For some characteristics, estimates using basic raking or post-stratification were shifted further away from high-quality population benchmark estimates.

Our results are consistent with a previous Australian study, which reported that full raking is more effective than post-stratification in reducing biases in estimates of health characteristics when Australian census data were used to weight the South Australian Monitoring and Surveillance System to the whole South Australian population [25]. For South Australia, they incorporated age, sex, place of residence, country of birth, marital status, education, dwelling status, employment status and number of people in the household to obtain raked weights, the first six of which were included for our full raking. This supports the value of including country of birth, marital status and education information in developing the weights, though in general the selection of appropriate characteristics depends on the sample and populations of interest, study aims, and available data. The raking method can incorporate more characteristics in the weights. The incorporation of these characteristics can result in more uncertainty in the weighted estimates, and this should be evaluated carefully, e.g. by considering the width of 95% confidence intervals or the margin of error (the distance from the prevalence estimate to each of the 95% confidence limits). Here, the differences in the margin of error between the weighted estimates from full raking and basic raking or post-stratification were minimal (on average, the margin of error for the 68 prevalence estimates included in Additional file 6 was 0.14% for post-stratification, 0.14% for basic raking, and 0.18% for full raking, thus increasing by < 0.1% for the latter).

Our results also align with a previous study [26], which weighted the 45 and Up Study sample using the same post-stratification approach as used here. That study compared weighted prevalence estimates for a wide range of characteristics to those from the NSW Population Health Survey, and also found that post-stratification had little impact on the estimated prevalence of most characteristics. For some characteristics, such as language other than English spoken at home, it slightly reduced the bias. However, post-stratification in their study and our basic raking and post-stratification, increased the estimated prevalence of indicators for high socioeconomic status such as private health insurance, higher educational attainment and higher household income, which are known to be over-represented in the sample [2]. By contrast, full raking resulted in higher weighted proportions for lower socioeconomic status, worse health, and riskier health behaviours. This suggests the post-stratification method may not be sufficient for correcting complex biases due to its inability to incorporate many characteristics.

Current smoking is a key risk factor for which basic post-stratification and basic raking did not improve under-representation. While full raking increased the prevalence estimate for current smokers in the 45 and Up Study, the estimate remained lower than in two respected population-based surveys. The under-representation of current smokers aligns with a lower unweighted estimate of lung cancer incidence for males, which was strongly but not completely eliminated by applying fully raked weights. This suggests the characteristics incorporated in the fully raked weights do not completely capture factors associated with smoking prevalence or lung cancer incidence for males in the 45 and Up Study.

Recent work using post-stratification to weight the UK Biobank participants to those in the Health Survey for England has also shown that lack of representativeness may distort associations between risk exposures and disease [27]. However, limitations of that work included potentially missing some sources of biases due to weighting to a non-representative survey and missing data leading to exclusion of 25% of UK Biobank participants, which may be problematic if data are not missing at random [28]. We suggest that if possible, full raking to census estimates, may be useful for large datasets such as those from the UK Biobank and All of Us, which have good representation of population groups with a wide range of characteristics.

To the best of our knowledge, our study is the first to show the impact of the raking approach to weight cancer incidence. We have demonstrated that full raking improves the generalisability of estimated cancer incidence obtained from a sample to the Australian population. Where the incidence of female breast and colorectal cancers in the sample was similar to that in Australia, weighting did not alter the SIRs. This provides proof-of-concept and suggests raking may also be useful to improve representativeness of cancer incidence in other contexts. While we focused on developing weights for the whole 45 and Up Study sample, for studies focusing exclusively on the cancer sub-population, developing weights based on the NSWCR data may be more appropriate as this dataset contains important characteristics such as spread of disease.

This study has some limitations. The 45 and Up Study does not provide sampling weights (also known as design weights), therefore we were unable to apply such weights to account for the original sampling strategy. However, the post-stratification and basic raking methods that we have evaluated would approximate the sampling weights by accounting for differences in age, sex and place of residence between the study participants and the NSW or Australian population, and we demonstrated that these two weighting approaches were not sufficient to improve representativeness of many prevalence estimates. The Census has very limited health information for the entire Australian population, and therefore we could not assess more in-depth raking approaches including additional characteristics. Although the NDSHS and ANHS were conducted at a national level and weighted to make their samples more representative of the general population, differences in the distribution of some characteristics such as employment status may indicate that they are not truly representative for some characteristics. The 45 and Up Study, NDSHS and ANHS are drawn from slightly different populations (for example, the NDSHS and ANHS excluded residents in non-private dwellings such as aged care facilities, whereas 45 and Up Study participants were drawn from the Medicare enrolment database and could include these residents), and this may contribute to differences in the prevalence estimates across the studies. The mode of data collection differed between the 45 and Up Study and ANHS, which could further contribute to differences in estimates. Moreover, some questionnaire items were comparable but not identical across all studies. Nonetheless, the comparisons between 45 and Up Study data and both surveys provide some insights on the impact of weighting. Finally, we used NSWCR cancer incidence data as a proxy for the Australian data, after demonstrating that NSW and Australian cancer incidence rates were very similar (Additional file 2).

Despite these limitations, this study has several notable strengths. First, we used the 45 and Up Study, which is the largest cohort study in Australia covering a wide range of health and lifestyle characteristics. Second, our study compared the prevalence of a wide range of key health and sociodemographic characteristics to two large population-based surveys. We also examined weights to match the 45 and Up Study sample to both the NSW and Australian populations, and considered two sets of characteristics for raking. Third, linkage to population-wide cancer registry data enabled us to examine the impact of weighting on cancer incidence estimated from the sample.

## Conclusion

In conclusion, the findings from this study may be particularly useful for studies using the 45 and Up Study data aiming to generalise the estimated prevalence of exposures to the NSW or Australian populations. The available linkage of this prevalence data to administrative health data provides richer insights on joint associations than examining survey or health registry data alone. Additionally, the findings are potentially useful for researchers needing to extrapolate the prevalence of exposures from other health survey data. For example, multiple models in the Cancer Intervention and Surveillance Modelling Network (CISNET) simulate the risk of colorectal cancer based on multiple risk factors including obesity and smoking, and thus require representative survey estimates for the joint distribution of these risk factors [29–31]. Raking may be a useful tool for improving the generalisability of the estimated prevalence of exposures or diseases from surveys to the general population.

## Supplementary Information

**Additional file 1.** Table showing harmonisation of categories for characteristics across the 45 and Up Study and survey questionnaires. A. Harmonisation of categories for 45 and Up Study and ABS Census. B. Harmonisation of categories for 45 and Up Study, NDSHS and ANHS.

**Additional file 2.** Figure showing comparison of age-standardised incidence rates for cancers of the lung, colorectum, prostate and breast including death certificate only and people with multiple cancers for New South Wales and Australia, using data from Cancer Data in Australia, 1982-2016. Age-standardised using the Australian population in 2001.

**Additional file 3.** Development of raking weights.

**Additional file 4.** Calculation of standardised incidence ratios.

**Additional file 5.** Table showing 45 and Up Study participants’ characteristics (2006-2009) used for the fully raked weights, percentages before weighting, as well as after applying post-stratification weights, basic raked weights and fully raked weights.

**Additional file 6.** Table showing 45 and Up Study participants’ socioeconomic, health and lifestyle characteristics before and after weighting, compared to NDSHS and ANHS.

**Additional file 7.** Figure of unweighted and weighted Standardised Incidence Ratios (SIRs) for cancers of the lung, colorectum, breast and prostate for the 45 and Up Study cohort compared to the NSW and Australian population, using NSW Cancer Registry data (2009-2013).

## Data Availability

The 45 and Up Study data used for this study are available from the Sax Institute. Data were accessed using the Secure Unified Research Environment (SURE), a secure computing environment that can be accessed remotely to analyse linked health data. Enquiries for data access can be made to the Sax Institute, but restrictions apply to their availability (see https://www.saxinstitute.org.au/our-work/45-up-study/for-researchers/ for details). The New South Wales Cancer Registry data on cancer incidence can be obtained by submitting a request to the Cancer Institute NSW, but restrictions apply to their availability (see https://www.cancer.nsw.gov.au/ for details).
