# Supplementary material for: Raking of data from a large Australian cohort study improves generalisability of estimates of prevalence of health and behaviour characteristics and cancer incidence
Source: BMC Med Res Methodol. 2022 May 14;22:140. doi: 10.1186/s12874-022-01626-5 (PMC9107206; doi:10.1186/s12874-022-01626-5)
Supplement: Supplementary file 1 — Additional file 1. Table showing harmonisation of categories for characteristics across the 45 and Up Study and survey questionnaires. A. Harmonisation of categories for 45 and Up Study and ABS Census. B. Harmonisation of categories for 45 and Up Study, NDSHS and ANHS. [file 12874_2022_1626_MOESM1_ESM.docx]

**ADDITIONAL FILES**

**Additional file 1.**

**Table showing harmonisation of categories for characteristics across the 45 and Up Study and survey questionnaires**

**A. Harmonisation of categories for 45 and Up Study and ABS Census.**

| **Characteristic** | **45 and Up Study baseline questionnaire** | **ABS Census 2006 questionnaire** |
| --- | --- | --- |
| **Educational attainment** | Q5. What is the highest qualification you have completed? | Derived from two questions: |
|  |  | Q27. What is the highest year of primary or secondary school the person has completed? |
|  |  | Q28. What is the level of the highest qualification the person has completed |
| No School Certificate | * no school certificate or other qualification | * did not go to school, year 8 or year 9 AND level of education inadequately described |
|  |  | * did not go to school, year 8 or year 9 AND level of education not stated |
|  |  | * did not go to school, year 8 or year 9 AND not applicable |
| School Certificate | * school or intermediate certificate | * year 10 or 11 AND not applicable |
|  |  | * year 10 or 11 AND level of education not stated |
|  |  | * year 10 or 11 AND level of education inadequately described |
| Trade/Certificate/Diploma | * trade or apprenticeship | * all categories AND advanced diploma and diploma level |
|  | * certificate or diploma | * all categories AND certificate level |
|  |  | * all categories AND graduate diploma and graduate certificate level |
| Higher School Certificate | * higher school or leaving certificate | * year 12 AND level of education inadequately described |
|  |  | * year 12 AND level of education not stated |
|  |  | * year 12 AND not applicable |
| University degree or higher | * university degree or higher | * all categories AND bachelor degree level |
|  |  | * all categories AND postgraduate degree level |
| **Region of birth** | Q7. In which country were you born? (Standard Australian Classification of Countries; SACC) | Q12. In which country was the person born? |
| Australia | * Australia | * Australia |
| NZ and Oceania | * New Zealand | * New Zealand |
|  | * Melanesia | * Melanesia |
|  | * Micronesia | * Micronesia |
|  | * Polynesia | * Polynesia |
|  |  | * Antartica |
|  |  | * Oceania and Antartica |
| Asia | * North-East Asia | * North-East Asia |
|  | * South-East Asia | * South-East Asia |
|  | * Central and South Asia | * Central and South Asia |
| UK and Ireland | * United Kingfom | * United Kingfom |
|  | * Ireland | * Ireland |
| Europe | * Western Europe | * Western Europe |
|  | * Eastern Europe | * Northern Europe |
|  | * Eastern and Central Europe | * Southern and Eastern Europe |
|  |  | * North-West Europe |
| Other | * Middle East and North Africa | * North Africa and the Middle East |
|  | * Sub-Saharan Africa | * Americas |
|  | * North America | * Sub-Saharan Africa |
|  | * Central and South America |  |
| **Language other than English** | Q10. Do you speak a language other than English at home? | Q16. Does the person speak a language other than English at home? |
| No | * no | * no, English only |
| Yes | * yes | * yes |
| **Marital status** | Q13. What best describes your current situation? | Q6. What is the person's present marital status? |
| Never married | * single | * never married |
| Widowed | * widowed | * widowed |
| Divorced | * divorced | * divorced |
| Separated | * separated | * separated but not divorced |
| Married | * married, defacto/living with a partner | * married |

**B. Harmonisation of categories for 45 and Up Study, NDSHS and ANHS**

| **Characteristic** | **45 and Up Study baseline questionnaire** | **NDSHS 2007 and 2010 questionnaire** | **ANHS 2007 questionnaire** |
| --- | --- | --- | --- |
| **Alcohol intake (drinks/week**) | Q12. About how many alcoholic drinks do you have each week? one drink=a glass of wine, middy of beer or nip of spirits |  | ALKWKEC. Estimated total weekly consumption (in mls) |
| Non-drinker (< 1) | * < 1 |  | * < 1 |
| 1 to 3.5 | * 1 to 3.5 |  | * 1 to 3.5 |
| > 3.5 to ≤ 7 | * > 3.5 to ≤ 7 |  | * > 3.5 to ≤ 7 |
| > 7 to ≤ 14 | * > 7 to ≤ 14 |  | * > 7 to ≤ 14 |
| > 14 to ≤ 28 | * > 14 to ≤ 28 |  | * > 14 to ≤ 28 |
| > 28 | * > 28 |  | * > 28 |
| Missing | * missing |  | * not applicable |
| **Body Mass Index (kg/m^2^)** | Derived from two questions: | BMI. Categories | BMBMICAT. Body mass index categories |
|  | Q3. How tall are you without shoes? cm or feet/inches |  |  |
|  | Q4. About how much do you weigh? kg or stone, lbs |  |  |
| Underweight (<18.5) | * underweight (< 18.5) | Underweight | * grade 3 thinness (< 16) |
|  |  |  | * grade 2 thinness (16 to < 17) |
|  |  |  | * grade 1 thinness (17 to < 18.5) |
| Normal Range (18.5 to <25) | * normal (18.5 to < 25) | Healthy weight | * normal range (18.5 to < 20) |
|  |  |  | * normal range (20 to < 25) |
| Overweight (25 to <30) | * overweight (25 to < 30) | Overweight but not obese | * grade 1 overweight (25 to < 30) |
| Obese (≥ 30) | * obese (≥ 30) | Obese | * grade 2 overweight (30 to < 40) |
|  |  |  | * grade 3 overweight (≥ 40) |
| Missing | * missing | Missing | * not stated |
|  |  |  | * not known |
| **DVA white or gold card** | Q50. Which of the following do you have? - Department of Veterans Affairs white or gold card |  | HIVETAFF. Type of Veterans Affairs treatment entitlement card |
| No | * no |  | * not applicable |
|  |  |  | * has other DVA card |
|  |  |  | * does not have a DVA card |
| Yes | * yes |  | * has white DVA card |
|  |  |  | * has gold DVA card |
| Missing | * missing |  | * not known if has a DVA card |
| **Employment status** | Q47. What is your current work status? | zz9. Which of the following best described your current employment status? | EMPSTABC. Labour force status |
| Employed | * in full time paid work | * self employed | * employed |
|  | * in part time paid work | * employed for wages |  |
|  | * partially retired |  |  |
|  | * self-employed |  |  |
| Unemployed | * unemployed | * unemployed and looking for work | * unemployed |
| Not in the labour force | * completely retired/pensioner | * home duties | * not in the labour force |
|  | * disabled/sick | * student |  |
|  | * doing unpaid work | * retired or on a pension |  |
|  | * studying | * unable to work |  |
|  | * looking after home/family |  |  |
| Missing | * other | * other | * not applicable |
|  | * missing | * not answered |  |
| **Ever diagnosed with asthma** | Q24. Has a doctor EVER told you that you have asthma |  | ASTHQ03 Whether still gets asthma (applies to persons who have ever had asthma) |
| No | * no |  | * not applicable |
| Yes | * yes |  | * still gets asthma |
|  |  |  | * does not still get asthma |
| Missing | * missing |  |  |
| **Ever diagnosed with diabetes** | Q24. Has a doctor EVER told you that you have diabetes |  | AGEDIABE. Age first told had diabetes or high sugar level (applies to persons who ever had diabetes) |
| No | * no |  | * not applicable |
| Yes | * yes |  | * less than 21 years |
|  |  |  | * 21-84 years |
|  |  |  | * 85 years and over |
| Missing | * missing |  | * not stated |
|  |  |  | * not known |
| **Fruit Consumption** | Q44. About how many serves of fruit or glasses of fruit juice do you usually have each day? A serve is 1 medium piece or 2 small pieces or 1 cup of diced or canned fruit pieces. |  | DIETQ6. How many serves of fruit do you usually eat each day? including fresh, frozen and tinned fruit. A serve is 1 medium piece, 2 small pieces or 1 cup chopped or canned fruit |
| Don't eat fruit | * I don’t eat fruit |  |  |
| < 2 serves per day |  |  |  |
| ≥ 2 serves per day |  |  |  |
| Missing | * missing |  |  |
| **Household Income** | Q46. What is your usual yearly household income before tax, from all sources? | zz17 Combined household annual income, before tax from all sources | Derived from EMPSTABC. Weekly household income multiplied by 52 |
| <$20,000 p.a | * less than $5,000 year | < $20,800 | * < $20,000 per year |
|  | * $5,000-$9,999 per year |  |  |
|  | * $10,000-$19,999 per year |  |  |
| $20,000-$39,999 p.a | * $20,000-$29,999 per year | $20,800 - $41,599 | * $20,000 to < $40,000 per year |
|  | * $30,000-$39,999 per year |  |  |
| ≥$40,000 p.a | * $40,000-$49,999 per year | $41,600+ | * ≥ $40,000 per year |
|  | * $50,000-$69,999 per year |  |  |
|  | * $70,000 or more per year |  |  |
| Missing | * I would rather not answer the question | Separate 'Prefer not to say' and missing | * not known |
|  |  |  | * no income |
| **K10 distress scale** | Q57. During the past 4 weeks, about how often did you feel? Kessler psychological distress scale calculated on scale of 0 to 50 | Kessler 10 scale level of psychological distress | MNKESSLR. Kessler 10 score. Kessler psychological distress scale calculated on scale of 0 to 50 |
| Well (0 to 19) | Well (0 to 19) | Well (0 to 19) | Well (0 to 19) |
| Mild (20 to 24) | Mild (20 to 24) | Mild (20 to 24) | Mild (20 to 24) |
| Moderate (25 to 29) | Moderate (25 to 29) | Moderate (25 to 29) | Moderate (25 to 29) |
| Severe (30 to 50) | Severe (30 to 50) | Severe (30 to 50) | Severe (30 to 50) |
| Missing | Missing | Missing | * not collected |
|  |  |  | * unable to determine |
| **Main type of milk** | Q42. Which type of milk do you mostly have? |  | Derived from: |
|  |  |  | DIETQ1. What is the main type of milk that you usually use? |
|  |  |  | MILKFATU. What is the fat content of the milk usually use? whole milk/regular/full cream; reduced fat e.g. low/lite/HiLo; skim e.g. skinny/shape/Fat free. |
| Whole milk | * whole milk |  | * cows milk AND whole milk |
| Reduced fat milk | * reduced fat milk |  | * cows milk AND whole milk |
| Skim milk | * skim milk |  | * cows milk AND skim |
| Soy milk | * soy milk |  | * soy milk |
| Other milk | * other milk |  | * evaporated or sweetened condensed |
|  |  |  | * other type of milk |
| I don't drink milk | * I don't drink milk |  | * does not drink milk |
| Missing | * missing |  | * don't know AND don't know |
|  |  |  | * not applicable AND not applicable |
| **Overall health** | Q31. In general, how would you rate your overall health? | B1. In general, wouLd you say your health is… | SF12Q2. In general would you say that your health is |
| Excellent | * excellent | * excellent | * excellent |
| Very Good | * very good | * very good | * very good |
| Good | * good | * good | * good |
| Fair | * fair | * fair | * fair |
| Poor | * poor | * poor | * poor |
| Missing | * missing | * not answered | * not applicable |
|  |  | * response not used |  |
| **Private Health Insurance** | Q50. Which of the following do you have? - Private Health Insurance with extras or without extras |  | HEALQ01. Whether currently covered by private health insurance |
| No | * no |  | * without private health insurance |
| Yes | * yes |  | * with private health insurance |
| Missing | * missing |  | * not applicable |
|  |  |  | * not known |
| **Smoking status at baseline** | Q11. Have you ever been a regular smoker? | D7. Have you ever smoked on a daily basis? | SMKSTAT Smoker status |
| Current smoker | * yes and yes to regular smoker now | * yes I smoke daily now | * current smoker daily |
|  | * yes and no to regular smoker now but age stopped smoking within 1 year of age at baseline |  | * current smoker weekly (at least once a week but not daily) |
| Former smoker | * yes and no to regular smoker now | * yes, I used to smoke daily, not now | * ex-smoker |
| Never smoker | * no | * no, never smoked daily | * never smoked |
| Missing | * missing | * not answered | * not applicable |
| **Smoking intensity at baseline** | Derived from two questions: | Derived from two questions: |  |
|  | Smoking status at baseline classification (derived) | D7 Have you ever smoked on a daily basis |  |
|  | Q11. How much do you/did you smoke on average each day? (total cigs calculated as sum of cigarettes per day and pipes/cigars per day) | D13. Number of manufactured cigarettes smoked per day |  |
|  |  | D14. Number of roll-your-own cigarettes smoked per day |  |
|  |  | D15. Number of cigars or pipes smoked per day |  |
| Current smoker (20+ cigs) | * current smoker and 20 cigarettes or more per day |  |  |
| Current smoker (<20 cigs) | * current smoker and 0-20 cigarettes per day |  |  |
| Former smoker | * former smoker |  |  |
| Never smoker | * never smoker |  |  |
| Missing | * missing |  |  |
| **Smoking duration** | Derived: For former smoker, it is calculated as | Derived: For former smoker, it is calculated as D8-D9. For current smoker, it is calculated as age at survey-D9 | SMOKEAGEDE Duration of daily smoking (years) |
|  | Q11. age stopped smoking minus age started smoking. For current smokers, it is calculated as age at baseline minus age started smoking |  |  |
| < 6 years | * < 6 years |  |  |
| 6-10 years | * 6-10 years |  |  |
| 11-19 years | * 11-19 years |  |  |
| 20-29 years | * 20-29 years |  |  |
| 30-39 years | * 30-39 years |  |  |
| 40+ years | * 40+ years |  |  |
| Not applicable or missing |  |  |  |
| **Age started smoking** | Q11. How old were you when you started smoking regularly? | D9. About what age were you when you first start smoking daily? | SMOKEQ8E. How old were you when you first started to smoke regularly (that is, at least once a day)? |
| < 12 years | * < 12 years |  |  |
| 12-17 years | * 12-17 years |  |  |
| 18-24 years | * 18-24 years |  |  |
| 25-29 years | * 25-29 years |  |  |
| 30+ years | * 30+ years |  |  |
| Not applicable or missing | * never smoker or missing |  | * not applicable |
| **Age stopped smoking** | Q11. How old were you when you stopped smoking regularly? | D8. About what age were you when you stopped smoking daily? | SMOKE11E. How old were you when you stopped smoking regularly? |
| < 25 years | * < 25 years |  |  |
| 25-34 years | * 25-34 years |  |  |
| 35-44 years | * 35-44 years |  |  |
| 45-54 years | * 45-54 years |  |  |
| 55+ years | * 55+ years |  |  |
| Not applicable or missing | * never smoker, current smoker or missing |  | * not applicable |
| **Vegetable consumption** | Q43. About how many serves of vegetables do you usually have each day? A serve is half a cup of cooked vegetables or one cup of salad. |  | DIETQ4. How many serves of vegetables do you usually eat each day? including fresh, frozen and tinned vegetables. A serve is half cup of cooked vegetables or cooked legumes, 1 medium potato or 1 cup salad vegetables |
| Don't eat vegetables | * I don’t eat vegetables |  |  |
| < 5 serves per day | < 5 serves per day |  | < 5 serves per day |
| 5+ serves per day | 5+ serves per day |  | 5+ serves per day |
| Missing | * missing |  |  |
