# Supplementary material for: Raking of data from a large Australian cohort study improves generalisability of estimates of prevalence of health and behaviour characteristics and cancer incidence
Source: BMC Med Res Methodol. 2022 May 14;22:140. doi: 10.1186/s12874-022-01626-5 (PMC9107206; doi:10.1186/s12874-022-01626-5)
Supplement: Supplementary file 2 — Additional file 2. Figure showing comparison of age-standardised incidence rates for cancers of the lung, colorectum, prostate and breast including death certificate only and people with multiple cancers for New South Wales and Australia, using data from Cancer Data in Australia, 1982-2016. Age-standardised using the Australian population in 2001. [file 12874_2022_1626_MOESM2_ESM.docx]

**Additional file 2.**

**
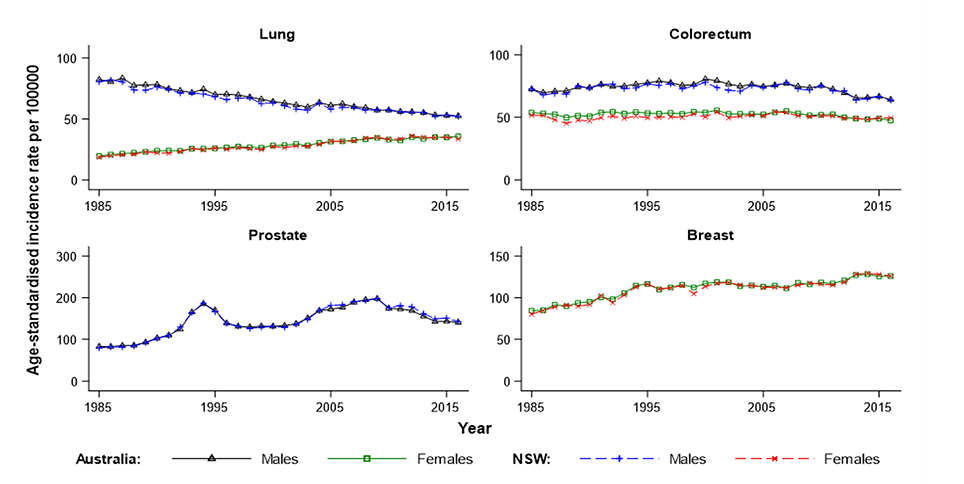
**

**Additional file 2.** Figure showing comparison of age-standardised incidence rates for cancers of the lung, colorectum, prostate and breast including death certificate only and people with multiple cancers for New South Wales and Australia, using data from Cancer Data in Australia, 1982-2016

Age-standardised using the Australian population in 2001
