# Supplementary material for: Raking of data from a large Australian cohort study improves generalisability of estimates of prevalence of health and behaviour characteristics and cancer incidence
Source: BMC Med Res Methodol. 2022 May 14;22:140. doi: 10.1186/s12874-022-01626-5 (PMC9107206; doi:10.1186/s12874-022-01626-5)
Supplement: Supplementary file 3 — Additional file 3. Development of raking weights. [file 12874_2022_1626_MOESM3_ESM.docx]

**Additional file 3.**

**Development of raking weights**

The application of the raking method using STATA’s ipfraking package has been outlined in detail ^1^ and an example of its application, to create the basic raked weight for the NSW population is shown here. Briefly, we applied the raking method to data from the 45 and Up Study sample using the variables sex, 5-year age group and place of residence to match the distribution of demographic data for the NSW population, obtained from the 2006 Australian Census ^2^.

Step 1. For each characteristic included in raking, create a variable (shown in upper case) that has the total number of people in the reference population (denoted as #) in each category (denoted as i):

***Create a new variable named “_ONE” that has the value 1 for each participant;

generate byte _ONE = 1

***Create a new variable named “CENSUS_NSW_SEX_i_” with 2 categories (i=1 or 2) representing male or female, that specifies the total number of people in the NSW population (denoted as #) in each category, obtained from the Census;

matrix CENSUS_NSW_SEX = (#census_nsw_sex_1_, #census_nsw_sex_2_)

matrix colnames CENSUS_NSW_SEX = 1 2

matrix coleq CENSUS_NSW_SEX = _ONE

***Let SAMPLE_45UP_SEX denote the variable name in the 45 and Up Study for variable sex.

matrix rownames CENSUS_NSW_SEX = SAMPLE_45UP_SEX

matrix list CENSUS_NSW_SEX

***Create a new variable named “CENSUS_NSW_AGE_i_” with 9 categories (i=1,2,….9) representing 5-year age groups (from 45-84 years, or ≥85), that specifies the total number of people in the NSW population (denoted as #) in each category, obtained from the Census;

matrix CENSUS_NSW_AGE = (#census_nsw_age_1_, #census_nsw_age_2_, #census_nsw_age_3_, #census_nsw_age_4_, #census_nsw_age_5_, #census_nsw_age_6_, #census_nsw_age_7_, #census_nsw_age_8_, #census_nsw_age_9_)

matrix colnames CENSUS_NSW_AGE = 1 2 3 4 5 6 7 8 9

matrix coleq CENSUS_NSW_AGE = _ONE

***Let SAMPLE_45UP_AGEGRP denote the variable name in the 45 and Up Study for age group.

matrix rownames CENSUS_NSW_AGE = SAMPLE_45UP_AGEGRP

matrix list CENSUS_NSW_AGE

***Create a new variable named “CENSUS_NSW_ARIA_i_” with 4 categories (i=1,2,3 or 4) representing major cities, inner regional, outer regional and remove or very remote, that specifies the number of people in the NSW population (denoted as #) in each category, obtained from the Census;

matrix CENSUS_NSW_ARIA = (#census_nsw_aria_1_, #census_nsw_aria_2_, #census_nsw_aria_3_, #census_nsw_aria_4_)

matrix colnames CENSUS_NSW_ARIA = 1 2 3 4

matrix coleq CENSUS_NSW_ARIA = _ONE

***Let SAMPLE_45UP_ARIA denote the variable name in the 45 and Up Study for place of residence.

matrix rownames CENSUS_NSW_ARIA = SAMPLE_45UP_ARIA

matrix list CENSUS_NSW_ARIA

*** Repeat the above process if including more variables for raking.

Step 2. Create the raking weights using the *ipfraking* procedure:

***Create a variable named “WEIGHT0” in the dataset that has the value 1 for all rows, indicating each 45 and Up Study participant represents one person in unweighted estimates;

generate WEIGHT0 = 1

***Create a new weight named ‘RAKINGWEIGHT_BASIC_NSW’ which weights 45 and Up Study data “WEIGHT0” to match the NSW distribution of sex, 5-year age group and remoteness of residence as defined in Step 1;

set more off

ipfraking [pw=WEIGHT0], generate(RAKINGWEIGHT_BASIC_NSW) ctotal(CENSUS_NSW_SEX CENSUS_NSW_AGE CENSUS_NSW_ARIA)

Step 3. Identify extreme outlying values to be removed in the created raking weights

***Output the median and interquartile range for ‘RAKINGWEIGHT_BASIC_NSW’;

tabstat RAKINGWEIGHT_BASIC_NSW, stat(median iqr min max)

***Calculate the lowest and highest value to be included in the weights, based on the formula median±6*(IQR), then exclude values lower than the ‘median-6*(IQR)’ and higher than the ‘median+6*(IQR)’;

ipfraking[pw=WEIGHT0], generate(RAKINGWEIGHT_BASIC_NSW_TRIM) ctotal(CENSUS_NSW_SEX CENSUS_NSW_AGE CENSUS_NSW_ARIA) trimloabs(median-6*(IQR)) trimhiabs(median+6*(IQR))

***Check the minimum and maximum values are within the lower and upper bound values;

tabstat RAKINGWEIGHT_BASIC_NSW_TRIM, stat(min max)

**References**

1. Kolenikov S. Calibrating Survey Data using Iterative Proportional Fitting (Raking). *The Stata Journal* 2014;**14**(1):22-59.

2. Australian Bureau of Statistics. TableBuilder. <https://www.abs.gov.au/websitedbs/censushome.nsf/home/tablebuilder> Accessed January 2019.
