# Supplementary material for: Raking of data from a large Australian cohort study improves generalisability of estimates of prevalence of health and behaviour characteristics and cancer incidence
Source: BMC Med Res Methodol. 2022 May 14;22:140. doi: 10.1186/s12874-022-01626-5 (PMC9107206; doi:10.1186/s12874-022-01626-5)
Supplement: Supplementary file 4 — Additional file 4. Calculation of standardised incidence ratios. [file 12874_2022_1626_MOESM4_ESM.docx]

**Additional file 4.**

**Calculation of standardised incidence ratios**

The standardised incidence ratio (SIR) is defined as

$$SIR=\frac{O}{E}$$

where

O denotes the observed number of incident cancer cases in the 45 and Up Study cohort, and

E denotes the expected number of cancer cases in the 45 and Up Study cohort obtained by applying the sex-age-specific incidence rates to the person-years-at-risk in the 45 and Up Study:

$$E=\sum_{i}^{A} {Rate}_{i}\times{PY}_{i}/100,000$$

${Rate}_{i}$ denotes the sex-age-specific incidence rates per 100,000 in the whole NSW population for 5-year age groups (from 45 to 84 years and ≥85), calculated as the number of incident cancer cases in the NSWCR population data divided by the corresponding number of people in the NSW population obtained from the ABS ^1^.

${PY}_{i}$ denotes the corresponding unweighted or weighted person-years-at-risk for i^th^ age group in the 45 and Up Study cohort.

As most participants were recruited from 2008 (Figure below), we calculated SIRs for the period 2009-2013.

The calculation of the confidence intervals for the standardised incidence ratios using the Fieller-based method explicitly assumes that the study sample is a subgroup of the reference population, so that:

${\left( O-E\times SIR \right)^{2}}/{Var\left( O-E\times SIR \right)}$ can be approximated by a $\chi^{2}$ distribution with 1 degree of freedom ^2^. 95% confidence intervals for the SIR are solutions to the quadratic equation:

$${SIR}^{2}\times E\times\left( E-\chi^{2} \right)-SIR\times\left( 2\times O\times E-{2\times O\times q\times\chi}^{2} \right)+O\times\left( O-\chi^{2} \right)=0$$

where *q* is the mean proportion of the reference population represented by the 45 and Up Study cohort averaged across age groups and weighted by the number of observed cancer cases in each age group ^2^.

The 45 and Up Study deliberately over-sampled individuals above the age of 80, so the proportion of the reference population represented by the study cohort was not constant. Consequently, we used a second approach to verify the robustness of the results, which does not assume the cohort is a subset of the population (but is limited by the assumption of independence between incidence in the study cohort and the reference population – see approach using the beta distribution in ^2^). The second approach resulted in very similar results and no change to any conclusions (data not shown).

**References**

1. Australian Bureau of Statistics. Australian Demographic Statistics, Table 51. Estimated Residence Population by single year of age, New South Wales. <http://www.abs.gov.au/ausstats/abs@.nsf/Latestproducts/3101.0Main%20Features1Sep%202018?opendocument&tabname=Summary&prodno=3101.0&issue=Sep%202018&num=&view>= Accessed February 2019.

2. Silcocks P. Estimating confidence limits on a standardised mortality ratio when the expected number is not error free. *J Epidemiol Community Health* 1994;**48**(3):313-7.

**
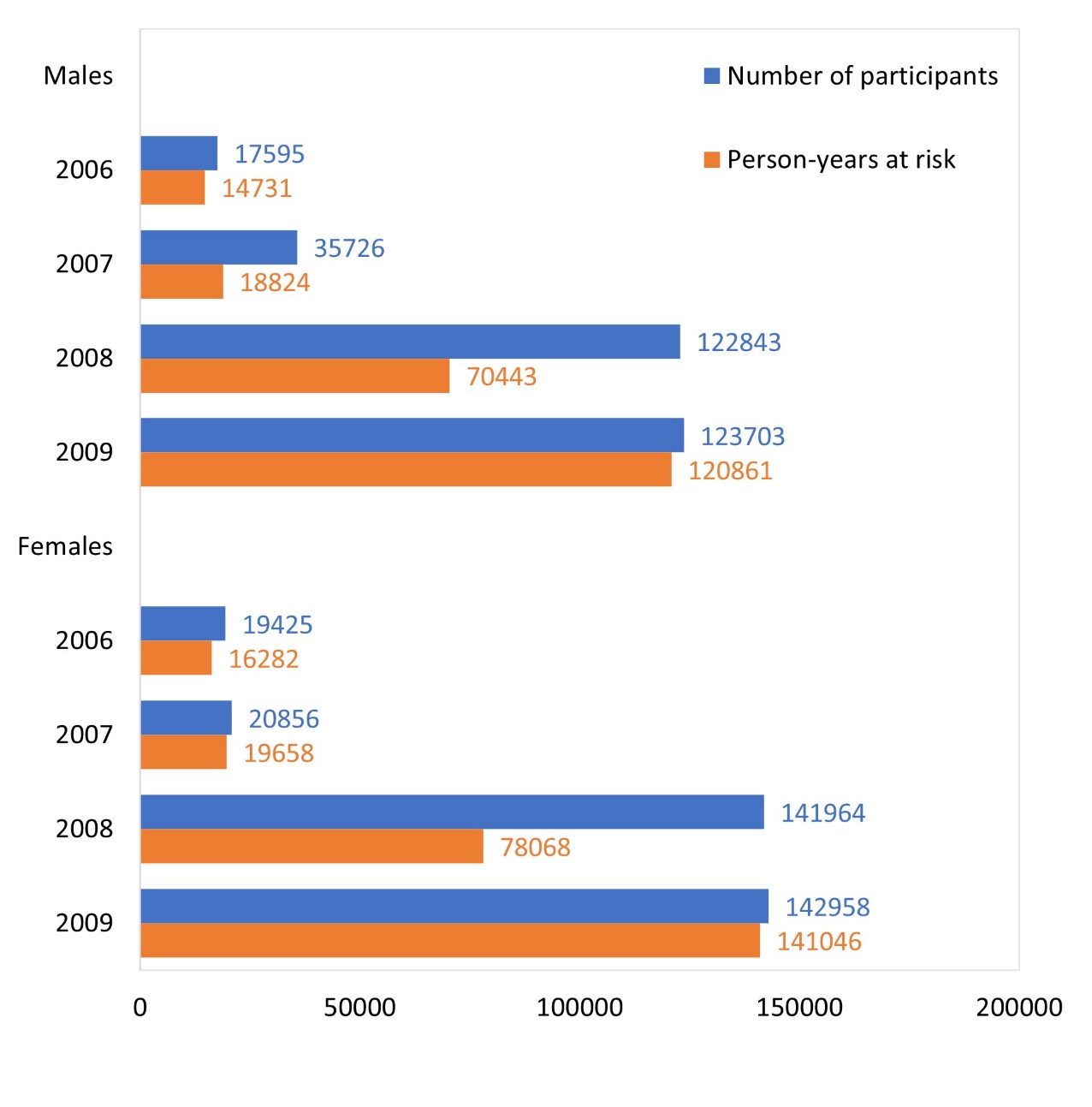
**

**Figure showing annual numbers of 45 and Up Study participants recruited and total person-years at risk by sex and year for 2006-2009.**
