# Supplementary material for: Raking of data from a large Australian cohort study improves generalisability of estimates of prevalence of health and behaviour characteristics and cancer incidence
Source: BMC Med Res Methodol. 2022 May 14;22:140. doi: 10.1186/s12874-022-01626-5 (PMC9107206; doi:10.1186/s12874-022-01626-5)
Supplement: Supplementary file 7 — Additional file 7. Figure of unweighted and weighted Standardised Incidence Ratios (SIRs) for cancers of the lung, colorectum, breast and prostate for the 45 and Up Study cohort compared to the NSW and Australian population, using NSW Cancer Registry data (2009-2013). [file 12874_2022_1626_MOESM7_ESM.docx]

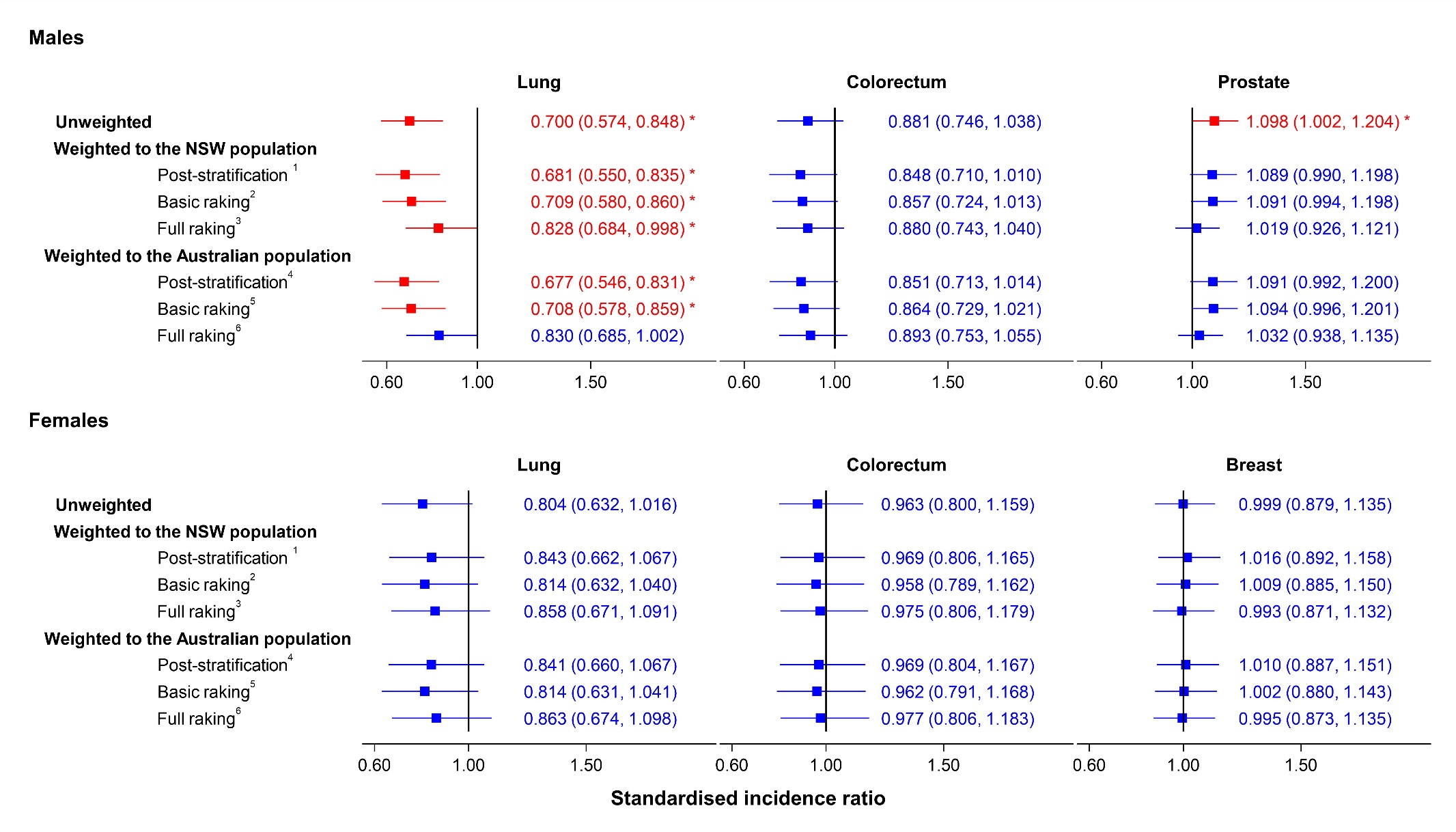


**Additional file 7.** Figure of unweighted and weighted Standardised Incidence Ratios (SIRs) for cancers of the lung, colorectum, breast and prostate for the 45 and Up Study cohort compared to the NSW and Australian population, using NSW Cancer Registry data (2009-2013).

1 Estimates after post-stratification based on the characteristics sex, age and place of residence and matching to the ABS Census 2006 data (restricted to the NSW population).

2 Estimates after basic raking based on the characteristics sex, age and place of residence and matching to the ABS Census 2006 data (restricted to the NSW population).

3 Estimates after full raking based on all characteristics listed in Table 1 and matching to the ABS Census 2006 data (restricted to the NSW population).

4 Estimates after post-stratification based on the characteristics sex, age and place of residence and matching to the ABS Census 2006 data for the whole Australian population.

5 Estimates after basic raking based on the characteristics sex, age and place of residence and matching to the ABS Census 2006 data for the whole Australian population.

6 Estimates after full raking based on all characteristics listed in Table 1 and matching to the ABS Census 2006 data for the whole Australian population.

* Statistically significant at 5% level
